# Influence of perylenediimide–pyrene supramolecular interactions on the stability of DNA-based hybrids: Importance of electrostatic complementarity

**DOI:** 10.3762/bjoc.10.164

**Published:** 2014-07-11

**Authors:** Christian B Winiger, Simon M Langenegger, Oleg Khorev, Robert Häner

**Affiliations:** 1 Department of Chemistry and Biochemistry, University of Bern, Freiestrasse 3, CH-3012 Bern, Switzerland

**Keywords:** DNA, hybridization, nucleic acids, perylenediimide, pyrene

## Abstract

Aromatic π–π stacking interactions are ubiquitous in nature, medicinal chemistry and materials sciences. They play a crucial role in the stacking of nucleobases, thus stabilising the DNA double helix. The following paper describes a series of chimeric DNA–polycyclic aromatic hydrocarbon (PAH) hybrids. The PAH building blocks are electron-rich pyrene and electron-poor perylenediimide (PDI), and were incorporated into complementary DNA strands. The hybrids contain different numbers of pyrene–PDI interactions that were found to directly influence duplex stability. As the pyrene–PDI ratio approaches 1:1, the stability of the duplexes increases with an average value of 7.5 °C per pyrene–PDI supramolecular interaction indicating the importance of electrostatic complementarity for aromatic π–π stacking interactions.

## Introduction

When two aromatic molecules are in close proximity they often have a tendency to interact non-covalently in a face-to-face stacking arrangement. Face-centered, parallel aromatic π–π stacking interactions have been studied and reviewed in great detail [1–5]. These interactions are especially important for polycyclic aromatic hydrocarbons (PAHs) [6–7]. The interaction is the result of solvophobicity, as well as van der Waals, electrostatic and charge transfer interactions that can lead to a thermodynamically favourable association [8]. It is an important interaction in biological systems, drug receptor interactions, materials sciences, and supramolecular chemistry [8–12]. Such interactions are strongly dependent on the electron density and distribution of the partners [2,9,13–16]. In particular, the interaction between electron-rich (donor) and electron-deficient (acceptor) aromatic rings results in stable aggregates [17–22].

In the DNA duplex, the interaction of the two complementary strands is governed mainly by aromatic π–π stacking interactions, hydrogen bonds, and electrostatic repulsion from the negatively-charged sugar phosphate backbone [10,23–28]. DNA can be regarded as an amphiphilic polymer in which aromatic residues are linked by negatively charged phosphodiester groups [29]. The importance of aromatic and hydrophobic factors for duplex stability was demonstrated by replacing the natural nucleobases by size expanded analogs [30–35].

A classic example of polymeric donor–acceptor complexes are the aedamers (*a*romatic *e*lectron *d*onor *a*cceptor oligo*mers*) pioneered by Iverson and coworkers [18,36–37]. They consist of face-to-face stacked electron-rich naphthalene and electron-poor naphthalenediimide (NDI) chromophores and belong to the broader area of foldamers [38].

**Figure 1:**
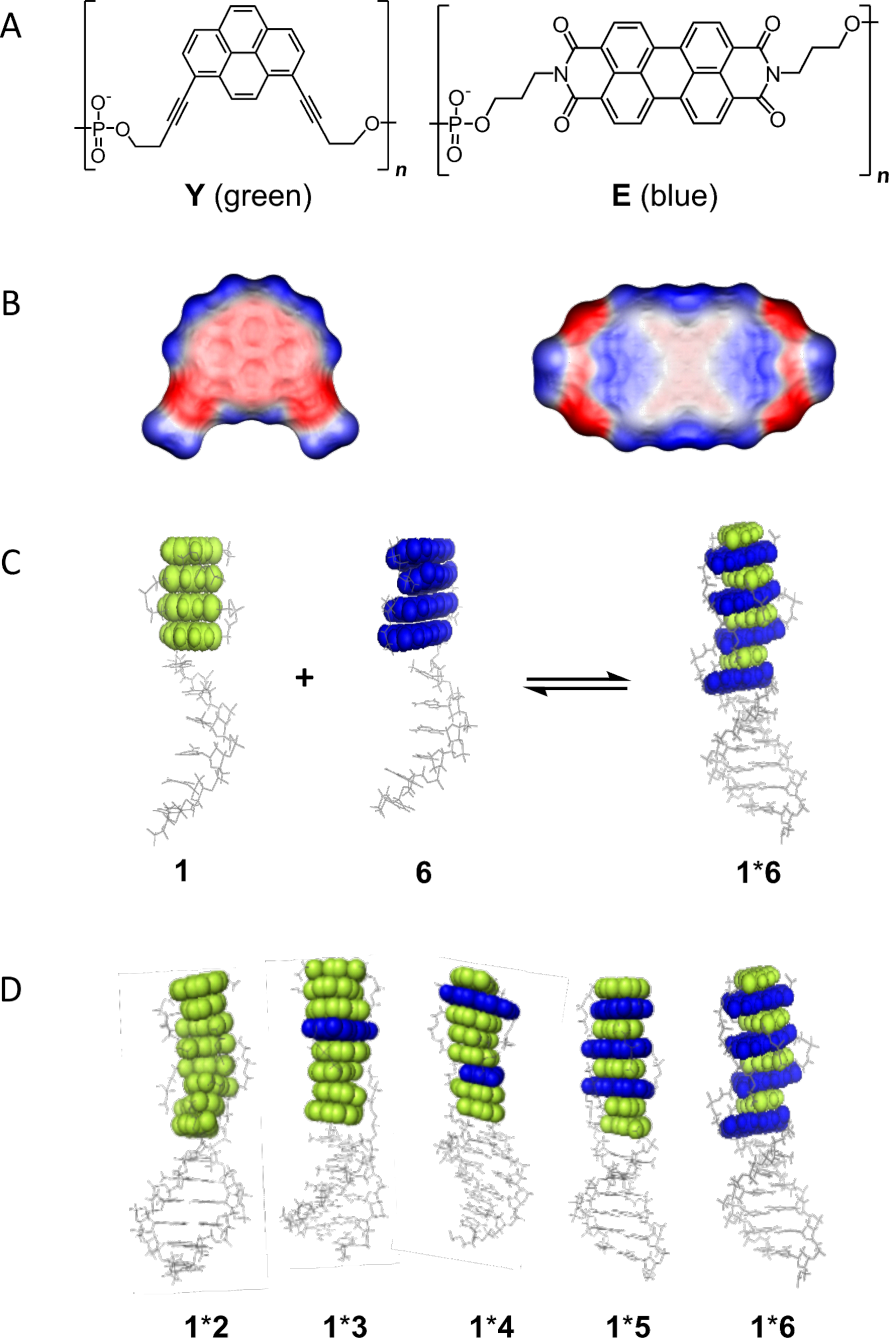
(A) Structures of 1,8-dialkynylpyrene (**Y**) and PDI (**E**); (B) illustration of the electrostatic potential surface of 1,8-diprop-1-ynylpyrene (left) and *N*,*N*’-dimethyl-PDI (right); (C) illustration of duplex formation with chimeric oligomers; (D) hybrids 1*2 to 1*6. The number of pyrene–PDI interactions increases from left to right.

DNA has been described as a molecular scaffold for arranging various types of chromophores [39–44]. Recently, we reported that oligoarenotides (oligomers with an alternating phosphodiester-aromatic hydrocarbon motif) exhibit similar structural properties as nucleic acids, and although the aromatic hydrocarbons cannot engage in any sort of Watson–Crick related hydrogen bonding, the individual strands interact via an interstrand stacking motif [45–48]. Herein we describe a series of DNA-based hybrids (Figure 1 and Table 1) containing electron-rich 1,8-dialkynylpyrenes (**Y**) and electron-poor perylenediimides (PDI, **E**).

**Table 1:** *T*_m_ values of the hybrids determined by thermal denaturation experiments.^a^

	Sequence	*T*_m_ (°C)	Number of pyrene–PDI interactions

Ref	5‘ GCGTTA 3‘ CGCAAT	13.0	
**1** **2**	5‘ GCGTTA **YYYY** 3‘ CGCAAT **YYYY**	50.5	0
**1** **3**	5‘ GCGTTA **YYYY** 3‘ CGCAAT **YYEY**	54.5	2
**1** **4**	5‘ GCGTTA **YYYY** 3‘ CGCAAT **YEYE**	58.5	4
**1** **5**	5‘ GCGTTA **YYYY** 3‘ CGCAAT **YEEE**	61.0	6
**1** **6**	5‘ GCGTTA **YYYY** 3‘ CGCAAT **EEEE**	64.5	7
**7** **2**	5‘ GCGTTA **EEEE** 3‘ CGCAAT **YYYY**	66.5	7
**7** **6**	5‘ GCGTTA **EEEE** 3‘ CGCAAT **EEEE**	52.0	0
**1** **7**	5‘ GCGTTA **YYYY** 5‘ GCGTTA **EEEE**	–^b^	n/a
**2** **6**	3‘ CGCAAT **YYYY** 3‘ CGCAAT **EEEE**	–^b^	n/a

^a^Conditions: oligomer conc. 2.5 μM single strand, 10 mM sodium phosphate buffer, pH 7.2, 0.1 M NaCl, absorption monitored at 260 nm; error ±0.5 °C; ^b^no transition observed (see Supporting Information File 1).

PDIs (Figure 1A) are some of the most widely studied organic chromophores [49–52]. Moreover, we have reported on the aggregation and stacking properties of 1,8- and 1,6-dialkynylpyrene [53–54]. Figure 1B shows the electrostatic potential surface of 1,8-diprop-1-ynylpyrene and *N*,*N*’-dimethyl-PDI. The former is considerably more electron-rich/higher electron density (red) than the latter, which is expected to favour an alternating aromatic π–π stacking arrangement of **E** and **Y** due to electrostatic complementarity.

We show herein that duplex formation by our chimeric DNA-oligoarenotide strands proceeds in a selective manner, the chromophores on opposite strands interdigitate and stack face-to-face in an organised controlled assembly.

## Results and Discussion

The principle of the system is illustrated in Figure 1. All oligomers are composed of a DNA part and a modified section containing a total of four PDIs (blue) and/or pyrenes (green). Oligomers **1**–**7** consisting of varying numbers of pyrene or PDI moieties covalently linked to complementary DNA strands were prepared by automated oligonucleotide synthesis using the previously described phosphoramidite pyrene [53] and PDI [55] building blocks.

The DNA stem acts as a supramolecular scaffold, and together with the flexible, negatively-charged phosphate linker allows the chromphores to adopt optimal conformations in aqueous solution and increases the solubility. The strands were hybridised in various combinations (Table 1), and their stability and photophysical properties were investigated. Since the DNA duplex is identical in all hybrids, differences in stability must originate from the modified section. The sequence of the modified part is changed in such a way that annealing of different strands leads to a varying number of pyrene–PDI stacking interactions. Strand **1** is common to all hybrids. The complementary strands **2**–**6** possess an increasing number of PDIs. Thus, in the resultant hybrids, the number of pyrene–PDI face-to-face stacking interactions also increases steadily from left to right, e.g., duplex 1*2 contains only pyrene–pyrene interactions, whereas duplex 1*6 has the maximum number pyrene–PDI interactions.

### Thermal denaturation experiments

**Figure 2:**
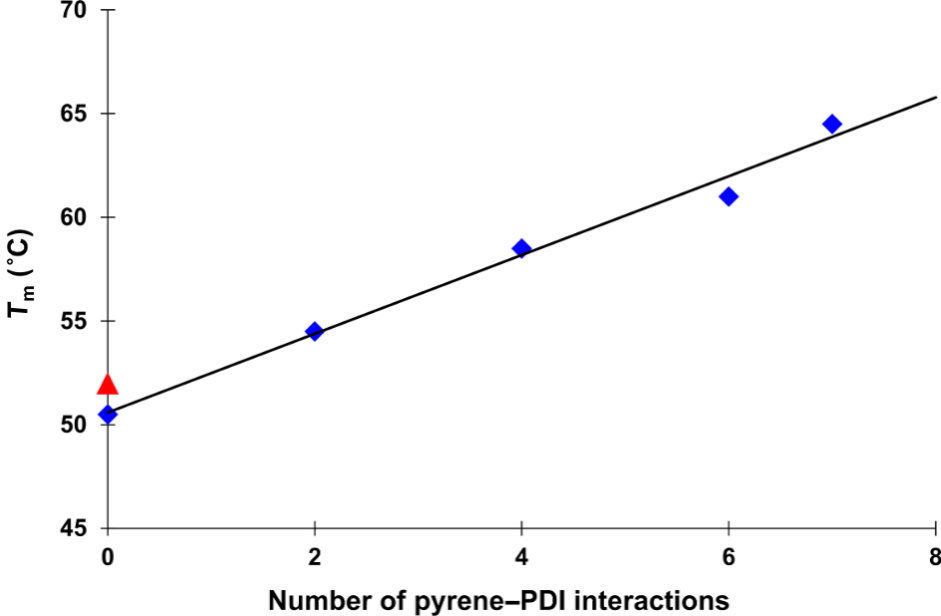
A plot of melting temperature (*T*_m_) versus the number of pyrene–PDI interactions for duplexes 1*2 to 1*6 (from left to right) presented in Table 1. The *T*_m_ was recorded at 260 nm; R^2^ = 0.987. The red triangle represents the *T*_m_ of the control hybrid 7*6.

Thermal denaturation experiments revealed a clear trend in duplex stability (Figure 2). The thermal stability correlates with the number of pyrene–PDI interactions [56] and increases linearly in the series. The melting temperature (*T*_m_) values are summarized in Table 1.

Hybrid 1*2 has a *T*_m_ of 50.5 °C which is 37.5 °C higher than the reference DNA duplex (*T*_m_ = 13 °C). Since hybrid 1*2 has seven pyrene–pyrene interactions, one of these interactions (Δ*T*_m/(Y−Y)_) contributes ≈ 5.4 °C to hybrid stability. Likewise, a value for Δ*T*_m/(E−E)_ = 5.6 °C is calculated for hybrid 7*6. The average contribution of a pyrene–PDI interaction can be calculated from the *T*_m_ difference (*T*_m_ = 51.5 °C) between 1*6 and the reference duplex. A value of Δ*T*_m/(Y−E)_ = 7.4 °C is obtained in this way. Hybrid 7*2 serves as a further control. In this duplex, the DNA and the modified parts of the two strands have been interchanged relative to 1*6. The *T*_m_ value of 7*2 is in the same range as 1*6 (66.5 and 64.5 °C, respectively), which translates into Δ*T*_m/(Y−E)_ = 7.7 °C. Thus, the average contributions to the hybrid stabilities are as follows: Δ*T*_m/(Y−E)_ ≈ 7.5 °C, whereas Δ*T*_m/(Y−Y)_ and Δ*T*_m/(E−E)_ ≈ 5.5 °C.

The results of electrostatic complementarity between an electron-rich pyrene and an electron-poor PDI is highlighted by the fact that duplexes with only pyrene or PDI are considerably less stable (Table 1, hybrids 1*2 and 7*6) than hybrids containing both types of aromatic compounds. Although the actual stability of such duplexes strongly depends on several parameters like, e.g., the geometry of the building blocks and the flexibility of the linkers, a general trend can be deduced from the thermal denaturation results that accounts for the above mentioned design of building blocks and sequences.

### UV–vis absorption spectroscopy

**Figure 3:**
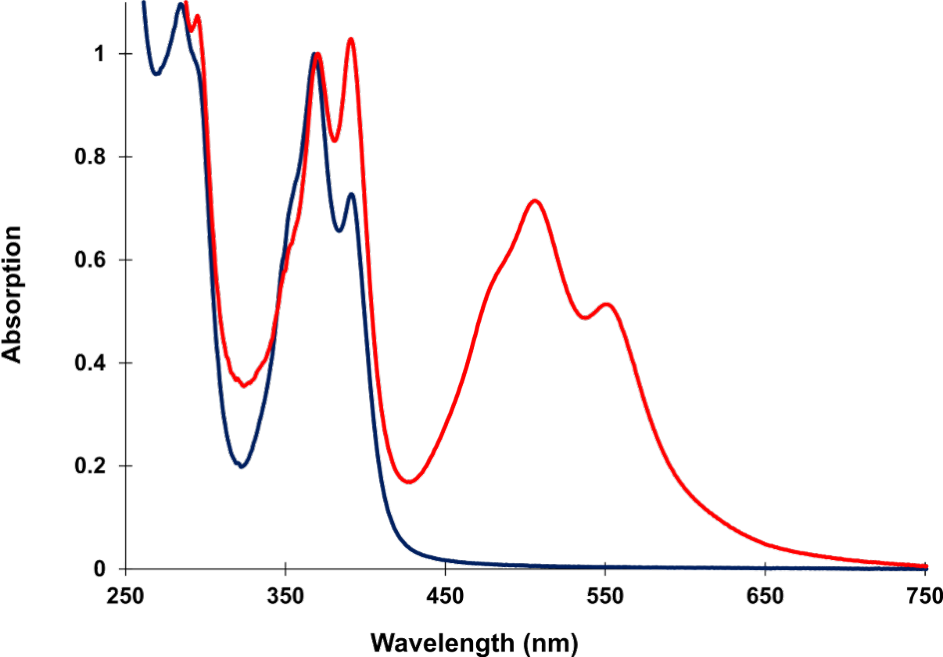
UV–vis absorption spectra (scaled) of duplexes 1*2 (blue) and 1*6 (red) at 20 °C. Conditions: see Table 1.

The stacking interactions of **Y** and **E** in the hybrids could be followed by UV–vis absorption spectroscopy. A significant change in the vibronic band ratio supports the model of an alternating interstrand interdigitation interaction between pyrene and PDI chromophores as proposed in Figure 1. In general, the vibronic band ratio of PAHs gives valuable information on the aggregation state of the molecules [57]. In a stack of only pyrenes (1*2) the vibronic band at 370 nm is higher than that at 390 nm (Figure 3), indicating that the pyrenes are stacked parallel and face-to-face. In contrast, in duplex 1*6 the intensity of the vibronic band 390 nm is higher indicating that the pyrenes are separated from each other by intercalating PDIs [58]. The same absorption behaviour is seen for the PDI vibronic band intensities.

**Figure 4:**
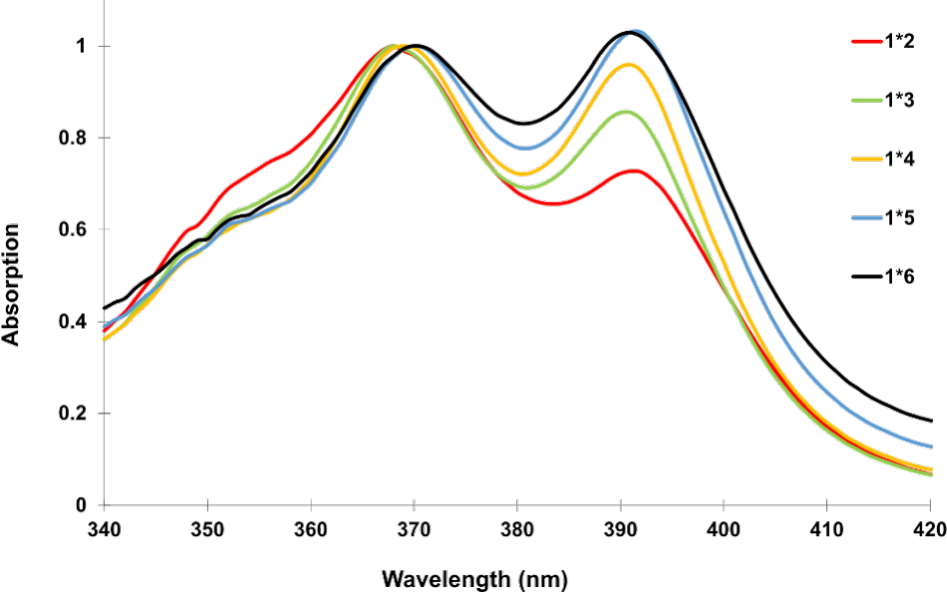
UV–vis absorption spectra (scaled) of duplexes 1*2 to 1*6 at 20 °C. Conditions: see Table 1.

Figure 4 focuses on the vibronic bands of pyrene’s S_0_→S_1_ absorption band in duplexes 1*2 to 1*6. An increasing number of PDIs in a stack leads to a stronger vibronic band at 390 nm.

**Figure 5:**
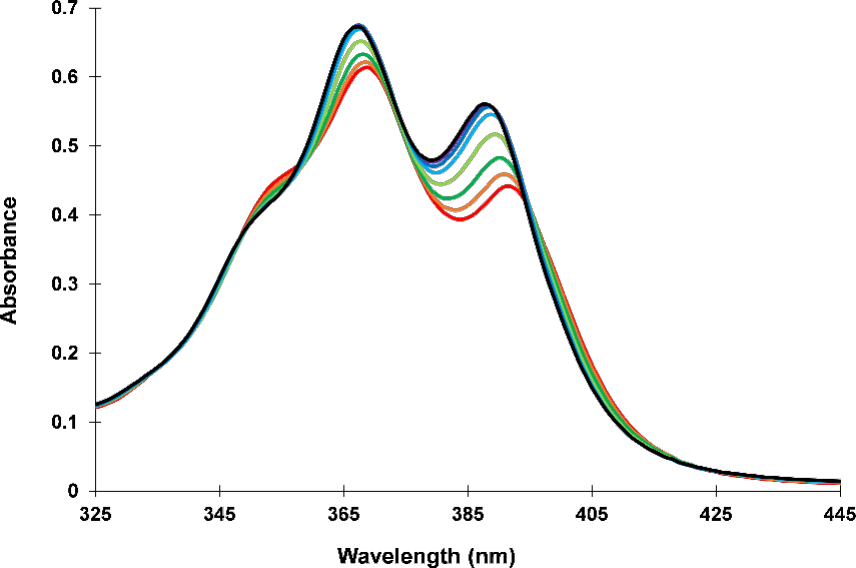
Temperature-dependent UV–vis absorption spectrum of 1*2. Conditions: see Table 1.

This is in stark contrast to the effect of thermally denaturing duplex 1*2 into two single strands (Figure 5). There, the vibronic band at 370 nm is always the highest indicating that the pyrenes are stacked even at 90 °C in the single strands. Such behaviour was also observed in chrysene-modified DNA [59].

### Fluorescence spectroscopy

**Figure 6:**
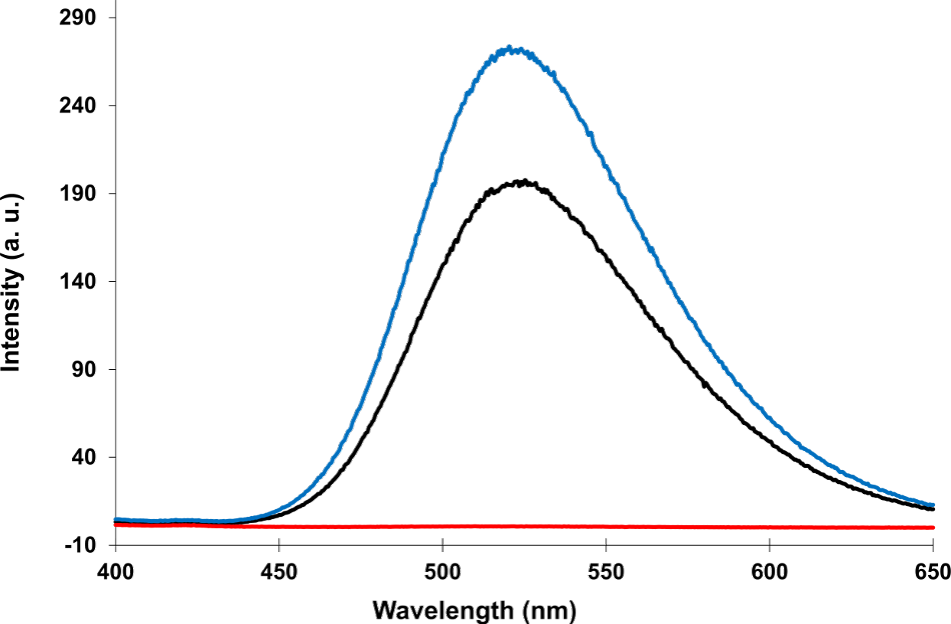
Fluorescence spectra of oligomer **1** (black), duplex 1*2 (blue) and duplex 1*6 (red) at 20 °C. Excitation: 370 nm. Conditions: see Table 1.

The interaction of two or more dialkynylpyrenes (**Y**) results in a pronounced excimer fluorescence [53]. Hybridization of single strands **1** and **2** increases the intensity of the excimer (Figure 6), whereas hybridization of single strands **1** and **6** results in an extinction of excimer fluorescence. Such behaviour was also observed in previous work and was explained by an alternating interdigitation interaction of the pyrene with the PDI building blocks [60].

### Gel migration experiments

**Figure 7:**
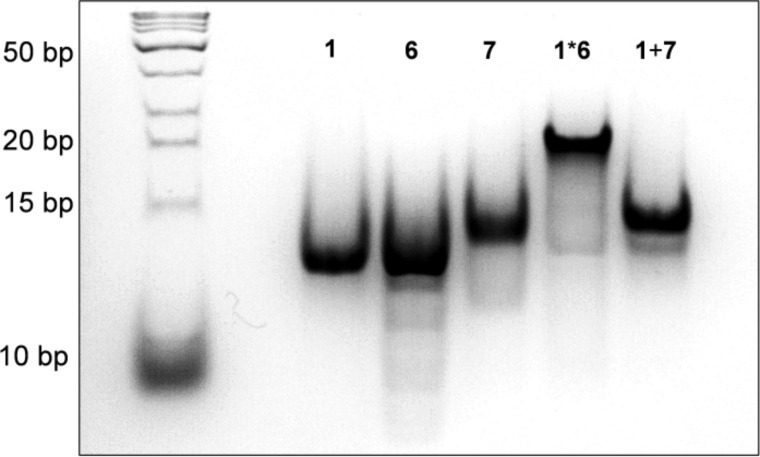
PAGE experiment. All oligomers were used in a total amount of 150 pmol in 10 mM sodium phosphate buffer, 100 mM NaCl and 10% loading buffer, 20% polyacrylamide gel with a 10% loading gel, 1 h 40 min, 4 °C, 170 V, 6 mA, 2 W. Left lane: DNA ladder.

The electrophoretic mobility of relatively small (<1000 kbp), linear DNA strands is inversely proportional to their molecular weight [61]. It serves as a reliable method to demonstrate the formation of double versus single stranded DNA structure. Therefore, the formation of defined short duplexes has been further investigated using polyacrylamide gel electrophoresis (PAGE) experiments. Oligomer single strands **1**, **6** and **7** migrate with the same velocity as the 13 bp reference (Figure 7). Strands **1** and **6**, however, form a duplex and thus have lower electrophoretic mobility, similar to an 18–20 bp reference. Oligomer **7** has the same DNA sequence as **6**, but with 4 PDI molecules instead of 4 pyrenes (see Table 1). Thus when combined, single strands **1** and **7** do not form a duplex due to having non-complementary DNA parts, and migrate on the gel like single strands **1**, **6**, and **7**. These results underline the importance of the complementary DNA segments in aligning the pyrene and PDI chromophores for optimal interaction.

## Conclusion

A series of DNA oligonucleotides functionalised with electron-poor perylenediimide (PDI, **E**) and electron-rich 1,8-dialkynylpyrene (**Y**) chromophores has been synthesized and their photophysical and thermal melting properties were investigated. UV–vis absorption and fluorescence spectra indicate an alternate, face-to-face, stacking of PDI and pyrene moieties. The DNA portion serves as an ideal scaffold to align the pyrene and PDI chromophores and to study their interaction in solution. The stability of the duplexes shows a clear dependence on the number of pyrene–PDI interactions within the duplex. As the pyrene–PDI ratio approaches 1:1, the stability of the duplexes increases with up to 7.5 °C per pyrene–PDI supramolecular interaction underlining the importance of electrostatic complementarity for aromatic π–π stacking interactions.

## Supporting Information

File 1 Detailed experimental procedures and supplementary spectroscopic data.
