# Supplementary material for: Influence of perylenediimide–pyrene supramolecular interactions on the stability of DNA-based hybrids: Importance of electrostatic complementarity
Source: Beilstein J Org Chem. 2014 Jul 11;10:1589–95. doi: 10.3762/bjoc.10.164 (PMC4142898; doi:10.3762/bjoc.10.164)
Supplement: File 1 — Detailed experimental procedures and supplementary spectroscopic data. [file Beilstein_J_Org_Chem-10-1589-s001.pdf]

Supporting Information

for

Influence of perylenediimide–pyrene supramolecular interactions on the stability of DNA-based hybrids

Christian B. Winiger, Simon M. Langenegger, Oleg Khorev and Robert Häner*

Address: Department of Chemistry and Biochemistry, University of Bern, Freiestrasse 3, CH-3012 Bern, Switzerland

*Corresponding author

E-mail: Robert Häner - robert.haener@dcb.unibe.ch

Detailed experimental procedures and supplementary spectroscopic data

Table of Contents

1. General procedures S2
2. HPLC purification S3
3. Mass spectrometry of oligomers **1**–**7** S7
4. UV–vis absorption spectra of oligomers **1**–**6** S8
5. UV–vis absorption spectra of hybrids **1*****2**–**1*****6** S8
6. UV–vis melting curves of the single strands and hybrids S9
7. UV–vis melting curves of hybrid **1*****7** with non-complementary DNA sequences S12
8. References S12

1. General procedures

All reagents and solvents were purchased from commercial suppliers and used without further purification. Water was taken from a MilliQ system. UV–vis spectra were measured on a Cary 100 Bio spectrophotometer. Fluorescence and excitation spectra were measured on a Cary Eclipse spectrofluorimeter. Mass-spectrometric data of the oligomers were obtained on Thermo Fisher LTQ Orbitrap XL using Nano Electrospray Ionization (NSI) in water/acetonitrile/triethylamine solution. The phosphoramidite building blocks perylenediimide (**E**) and 1,8-dialkynylpyrene (**Y**) were synthesized according to published procedures [1,2]. The oligomers **1**–**7** were prepared on an Applied Biosystems 394 DNA/RNA synthesizer. A standard cyanoethyl phosphoramidite coupling protocol was used beginning with nucleoside-loaded controlled pore glass (CPG) and Universal 3-CPG supports from Glen Research. Commercially available natural nucleoside phosphoramidites were dissolved in CH_3CN to yield 0.1 M solutions. The 1,8-dialkynylpyrene phosphoramidite was dissolved in 1,2-dichloroethane (0.1 M) and the perylenediimide phosphoramidite in dichloromethane (0.08 M). For the activation of the perylenediimide phosphoramidite a solution of 5-(ethylthio)-1*H*-tetrazole (ETT) in THF (0.3 M) was used. The CPG-bound oligonucleotides were cleaved and deprotected by treatment with 28–30% NH_4OH at 55 °C for 16 h. The supernatant was collected and the residue was washed three times with 1 mL EtOH/H_2O 1:1. Oligonucleotides synthesized on the Universal 3-CPG were cleaved using an adapted version (conc. aq. ammonia/methanol 1:2 v/v) of the provided cleavage and deprotection protocol (GlenResearch). After lyophilisation, the crude oligonucleotides were purified by reversed phase HPLC (Merck LiChroCART 250-4; LiChrospher 100, RP-18, 5 µm). A gradient starting with 0% up to 100% CH_3CN in 0.1 M aqueous triethylammonium acetate was set at a flow rate of 1.0 mL/min. The conditions for the HPLC purification are specified in the section 2 (see below). The purified oligonucleotides were dissolved in 1 mL H_2O. The concentration of the oligomers was determined using the pyrene band $\varepsilon_{370\ nm}$ = 36000 $M^{-1}cm^{-1}$ and the PDI band $\varepsilon_{545\ nm}$ = 23500 M^{-1}cm–$^{-1}$.

The PAGE experiments were performed using a 20% polyacrylamide/Bis solution (19:1, 5% C), prepared from a 40% stock solution (SERVA), and a 10% loading gel. The gel was run for 1 h 40 min at 4 °C, 170 V, 6 mA, 2 W. Around 150 pmol of oligomer was loaded in 10 mM sodium phosphate buffer and 100 mM NaCl. The samples were visualized using a Stains-all solution.

The graphical illustrations were prepared as follows: The electrostatic potential was visualized by ViewerLite 5.0 after geometry optimization (HyperChem 8.0 MM+). The schematic representations of the hybrids were obtained using HyperChem 8.0.

2. HPLC purification

The analytical runs were performed with the following time program.

Table S1: Time program used for analytical runs for the oligomers.

Time (min)	% acetonitrile
0.01	0
1.0	0
2.0	0
22.0	70
23.0	100
32.0	100
33.0	0
37.0	0

Table S2: Elution times and % acetonitrile for the oligomers purified.

Oligomer	Elution time (min)	% acetonitrile
1	20.9	66.6
2	21.1	67
3	17.0	53.75
4	15.0	47.41
5	14.62	45.84
6	15.3	48.3
7	15.4	48.49

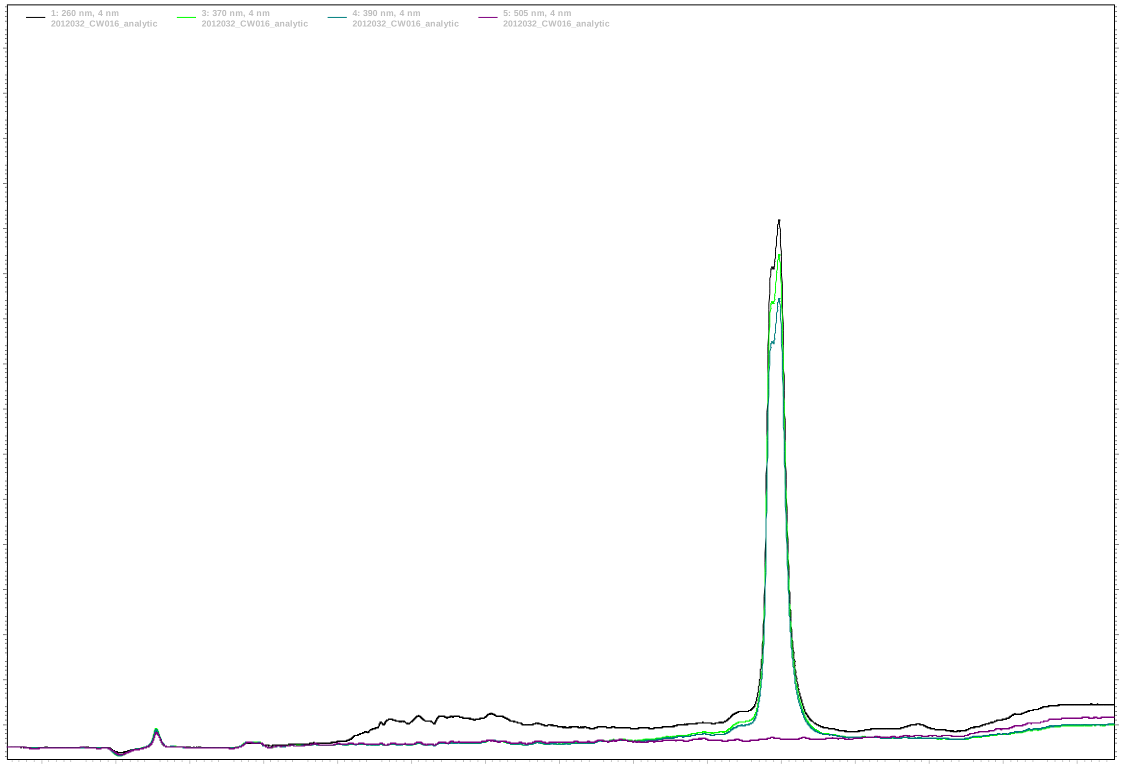

Figure S1: HPLC trace of oligomer **1** eluting at 20.9 min at 66.6% acetonitrile.

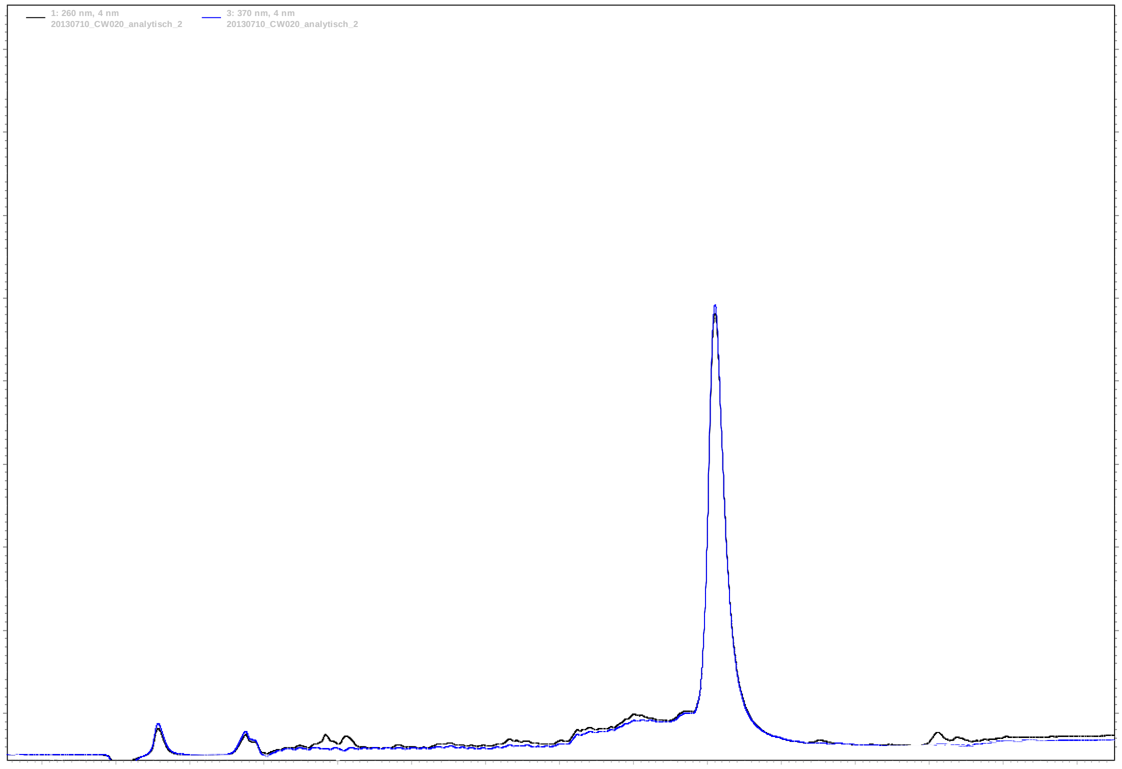

Figure S2: HPLC trace of oligomer **2** eluting at 21.1 min at 67% acetonitrile.

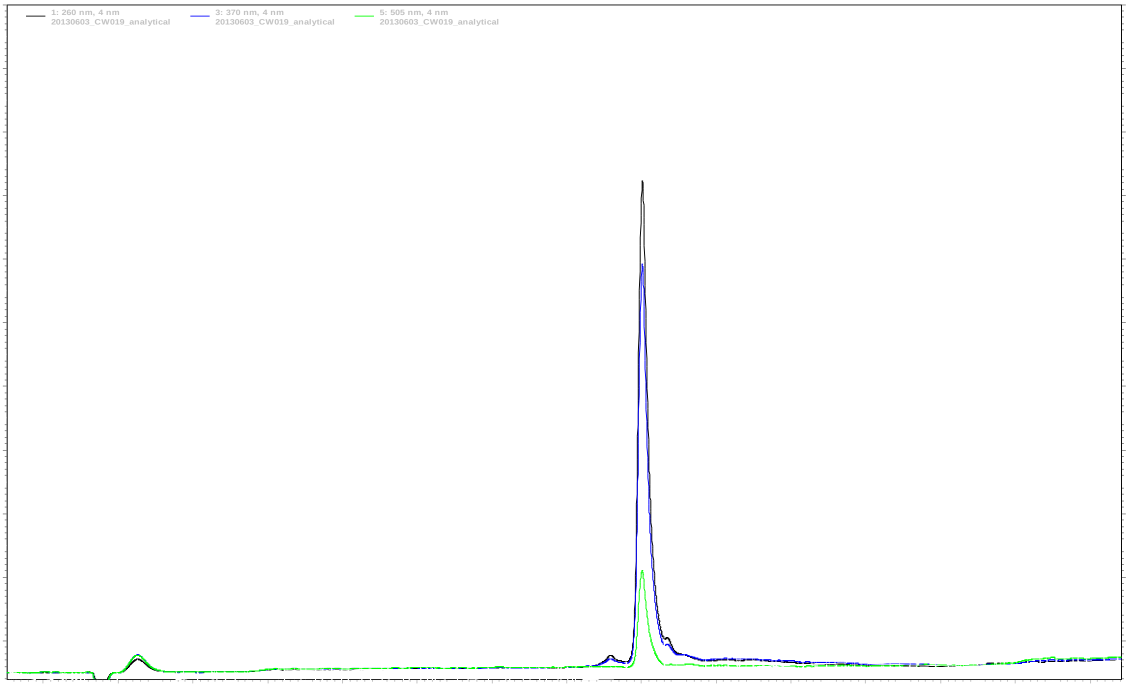

Figure S3: HPLC trace of oligomer **3** eluting at 17.0 min at 53.75% acetonitrile.

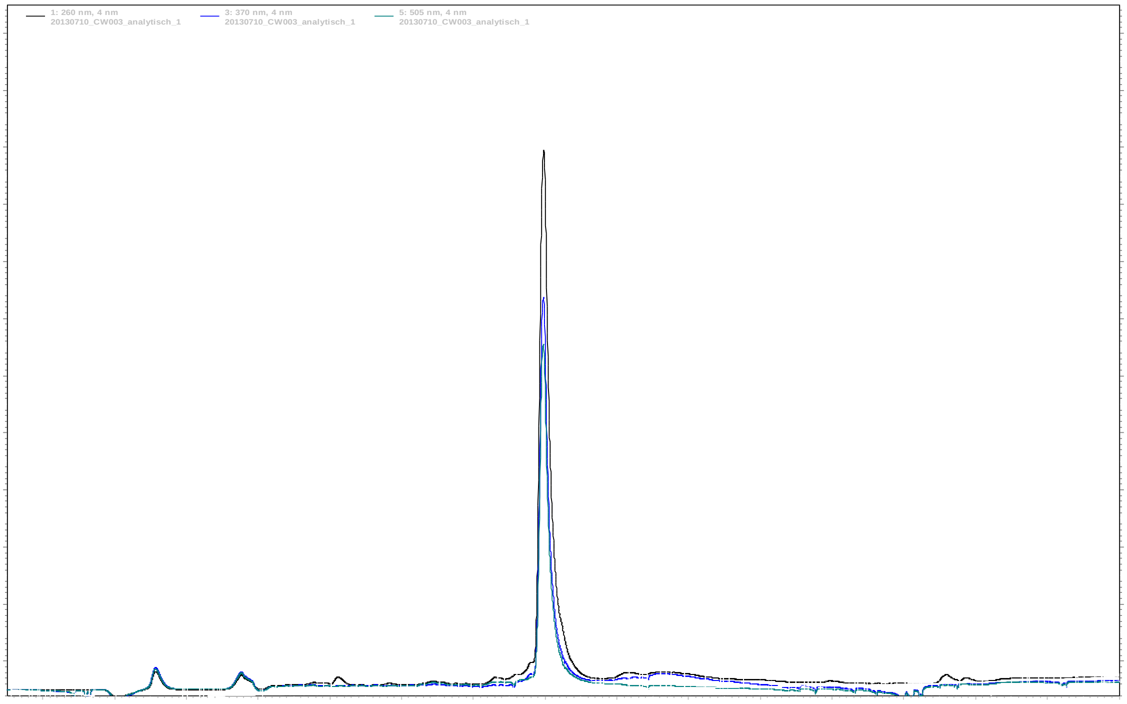

Figure S4: HPLC trace of oligomer **4** eluting at 15.0 min at 47.41% acetonitrile.

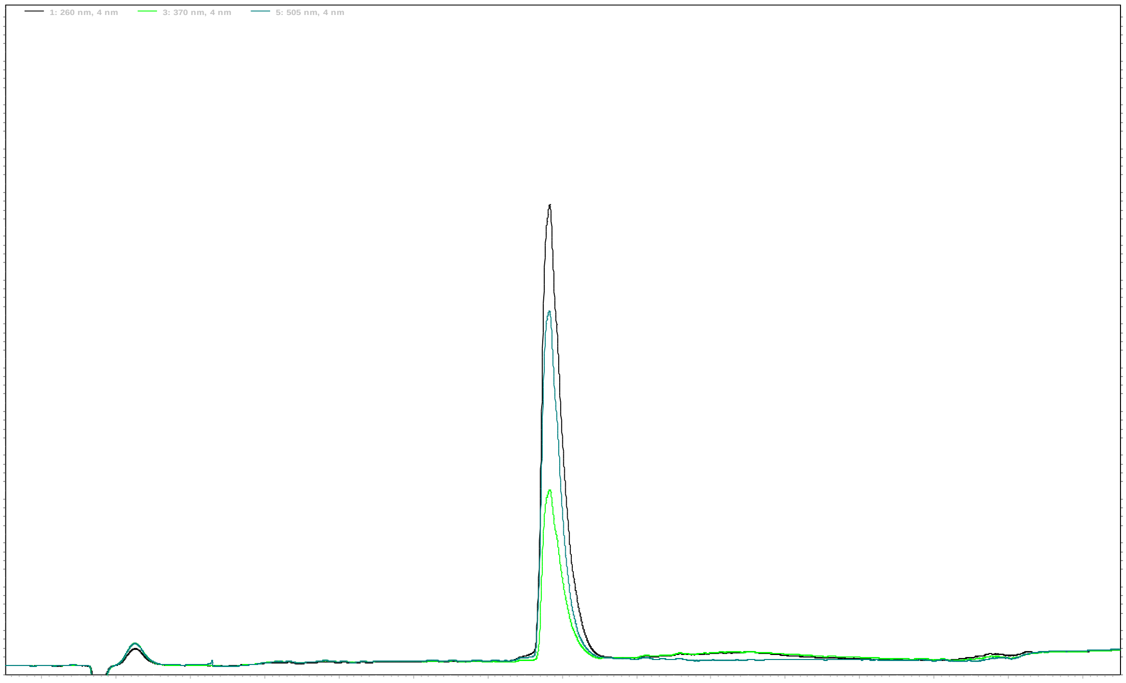

Figure S5: HPLC trace of oligomer **5** eluting at 14.62 min at 45.84% acetonitrile.

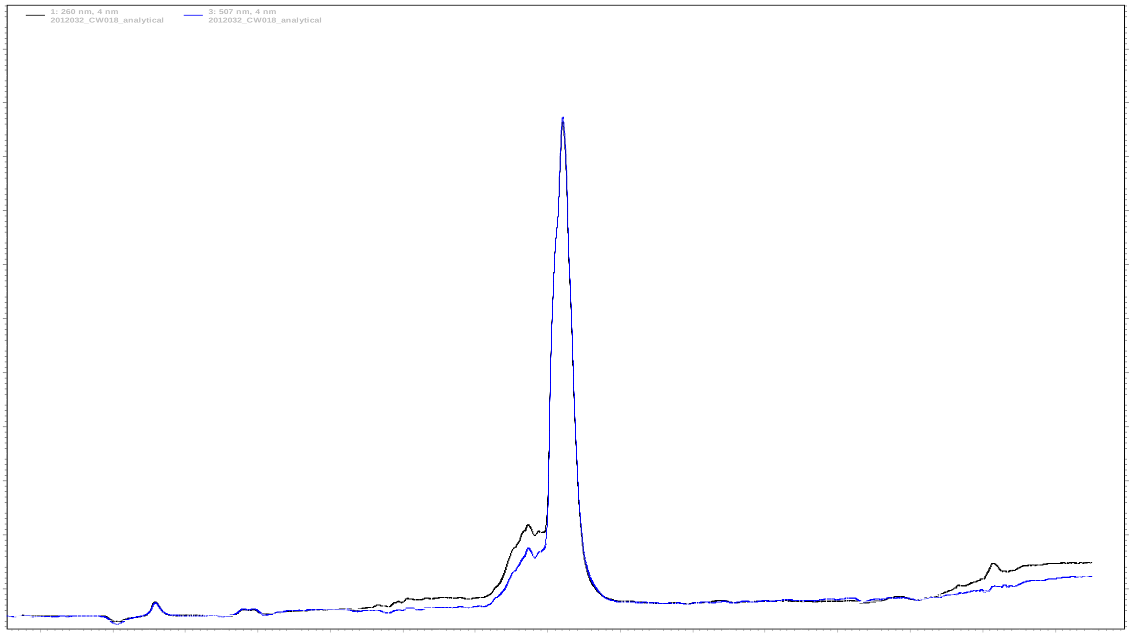

Figure S6: HPLC trace of oligomer **6** eluting at 15.3 min at 48.3% acetonitrile.

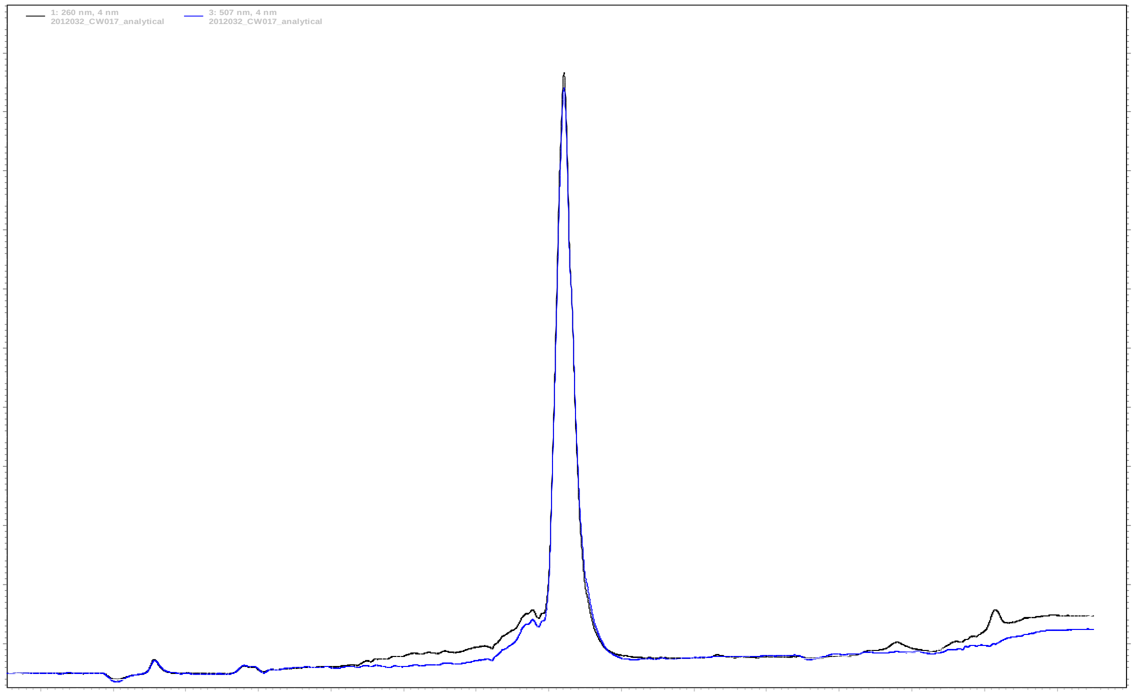

Figure S7: HPLC trace of oligomer **7** eluting at 15.4 min at 48.49% acetonitrile.

3. Mass spectrometry of oligomers 1–7

Oligomer	Sequence	Calculated	Found
**1**	5’ GCG TTA **YYY Y**	3407.6	3405.68
**2**	5‘ **YYY Y**TA ACG C	3376.6	3374.68
**3**	5‘ **YEY Y**TA ACG C	3544.6	3545.0
**4**	5‘ **EYE Y**TA ACG C	3712.6	3714.0
**5**	5‘ **EEE Y**TA ACG C	3880.7	3880.7
**6**	5‘ **EEE E** TAACG C	4048.7	4050.0
**7**	5‘ GCG TTA **EEE E**	4079.7	4080.0

4. UV–vis absorption spectra of oligomers 1–6

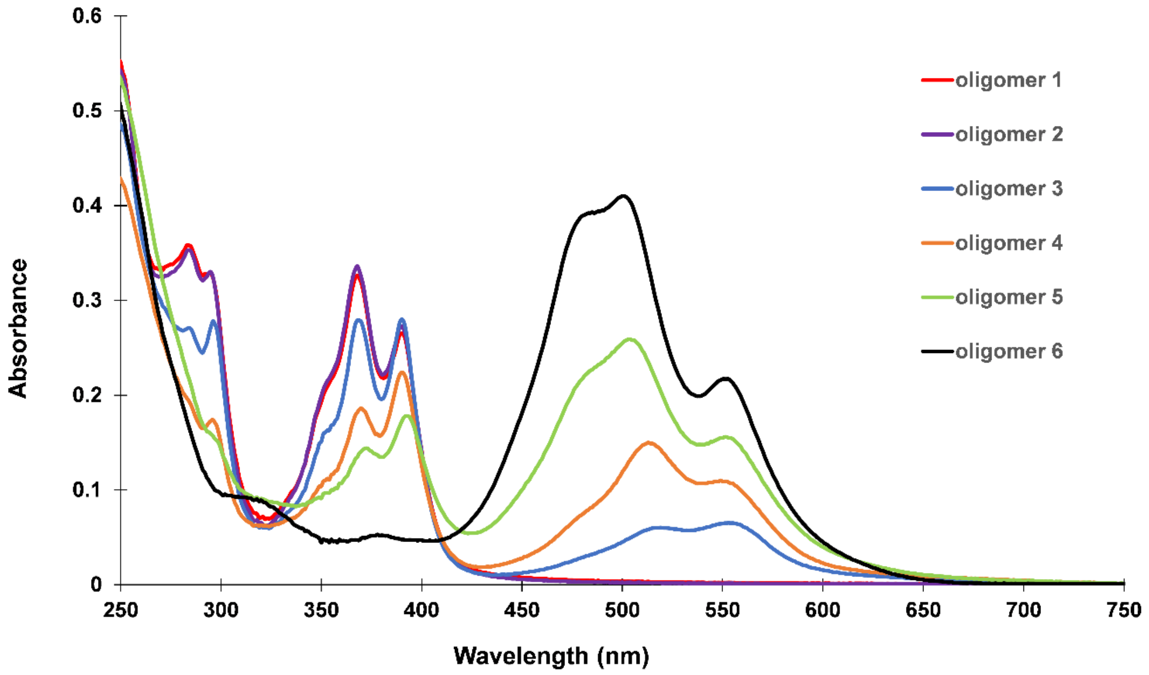

Figure S8: The samples were measured at 2.5 µM single strand oligomer in 10 mM sodium phosphate buffer, pH 7.2 and 100 mM NaCl at 20 °C.

5. UV–vis absorption spectra of hybrids 1*2–1*6

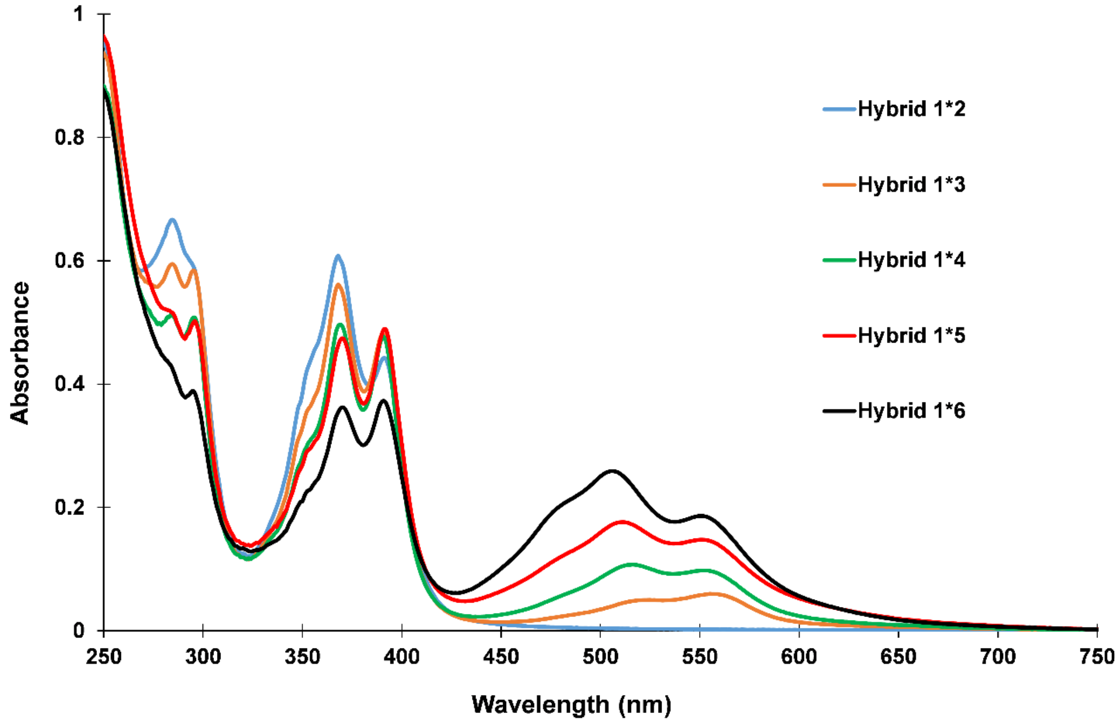

Figure S9: UV–vis absorption spectra of hybrids **1*2**–**1*6**. Conc. 2.5 µM each single strand in 10 mM sodium phosphate buffer, pH 7.2 and 100 mM NaCl at 20 °C.

6. UV–vis melting curves of the single strands and hybrids

Melting temperature experiments were carried out on a Varian-Cary-100 Bio spectrophotometer. A Cary probe temperature controller and Varian WinUV software were used. Samples were measured at a concentration of 2.5 µM (each single strand) in 10 mM sodium phosphate, pH 7.2 and 100 mM NaCl. The T_m was recorded in a cooling (ramp 1) – heating (ramp 2) – cooling (ramp 3) mode using 0.3 °C/min, monitoring at 260 nm unless otherwise stated. Calculation of the T_m was performed by fitting the melting curve with a polynomial fit (m = 9) and taking the first derivative. The 2nd ramp is evaluated.

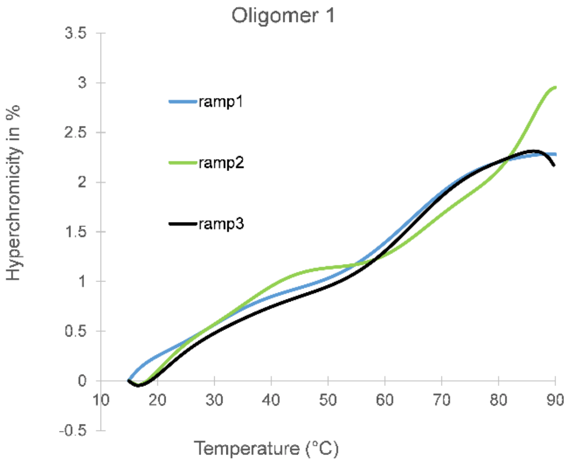

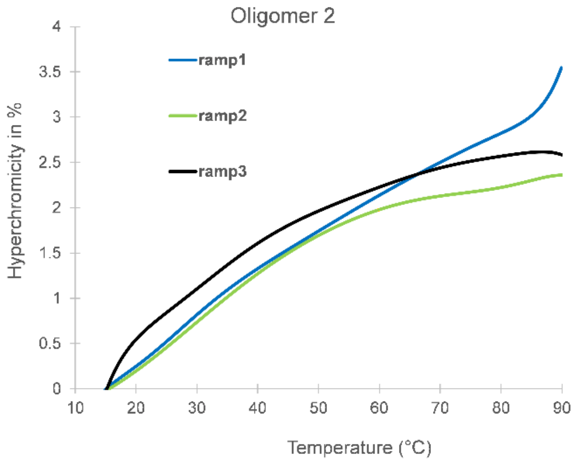

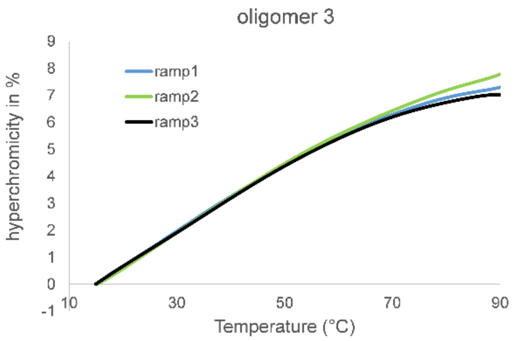

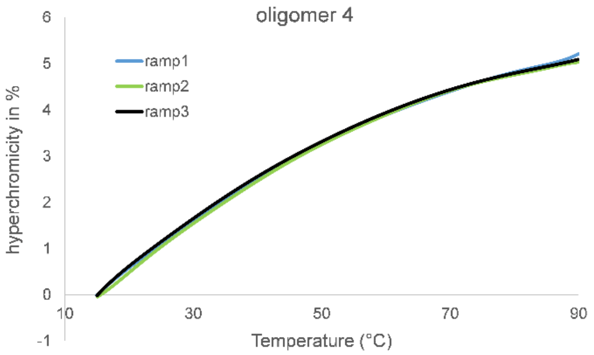

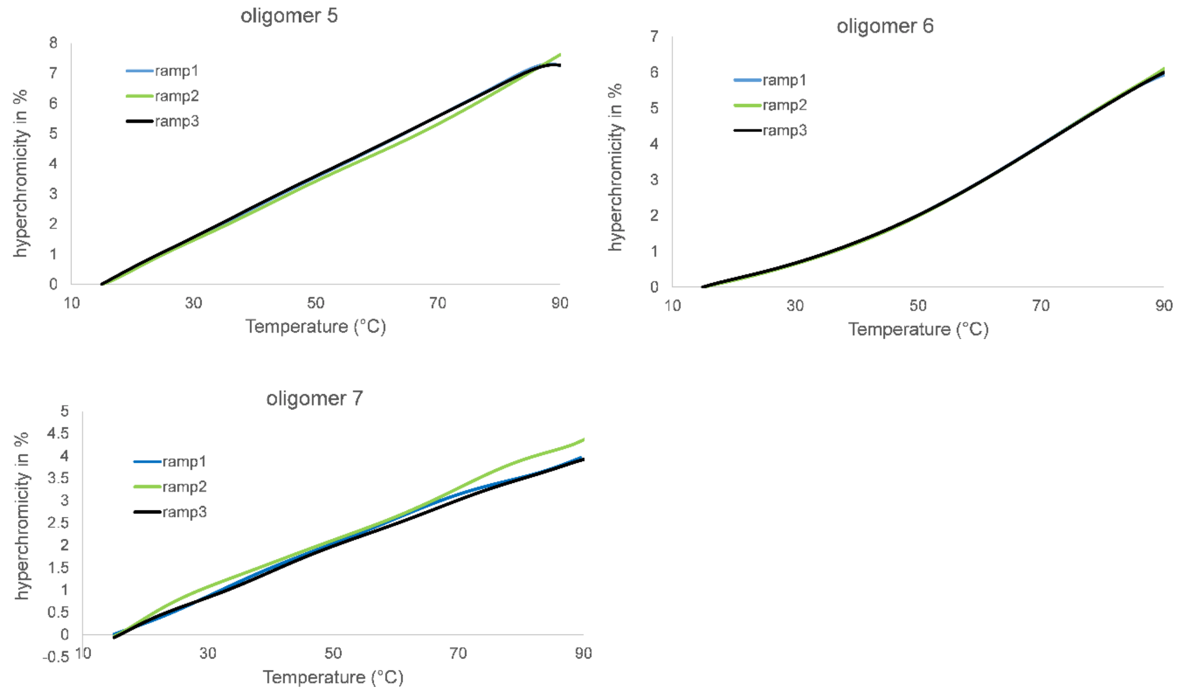

Figure S10: Temperature-dependent absorbance of the oligomer single strands**1**–**7**.

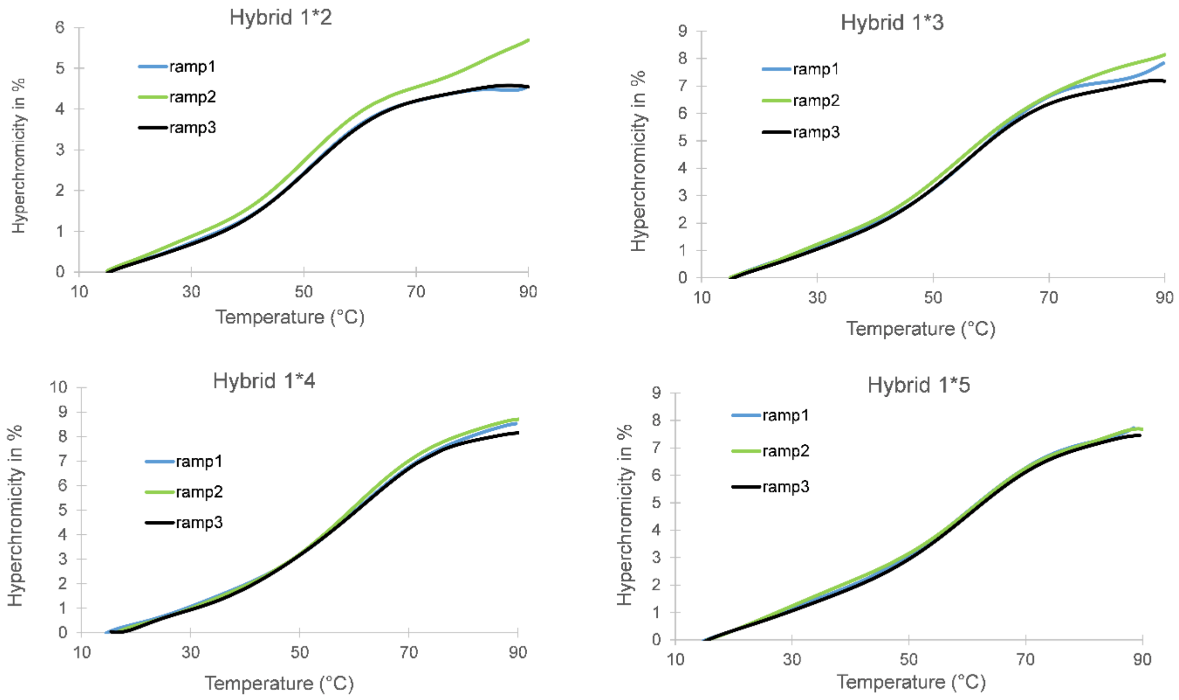

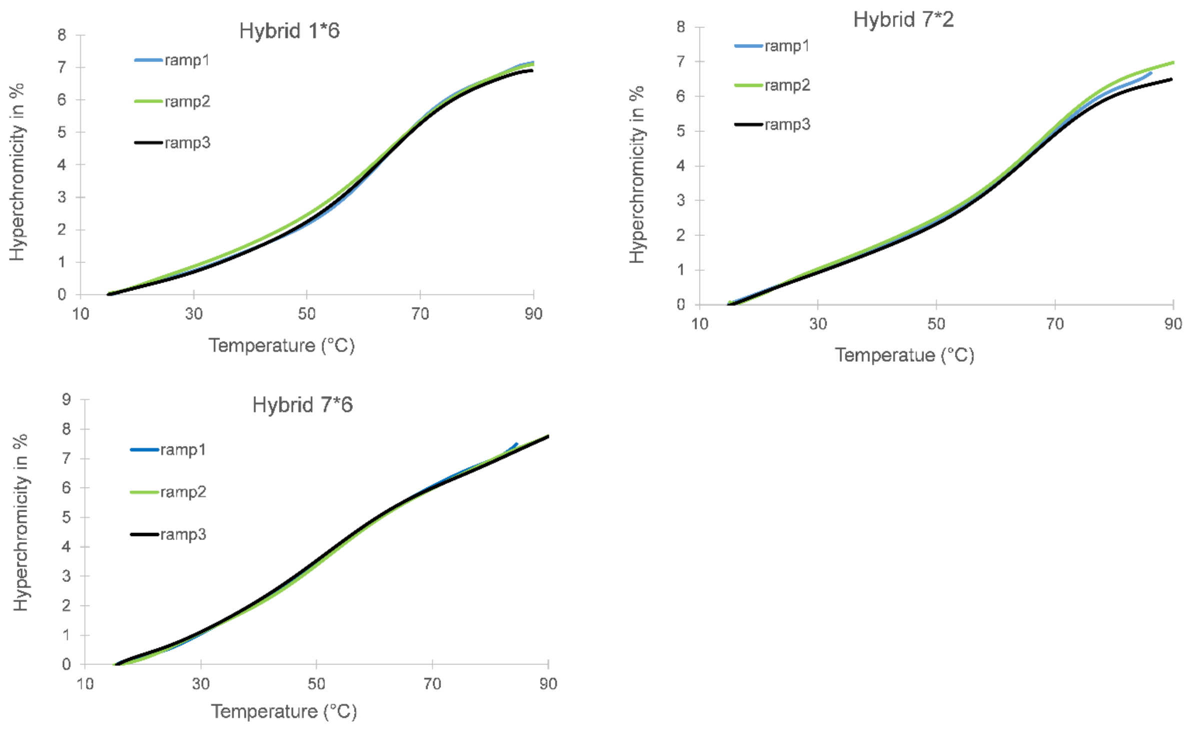

Figure S11: Temperature-dependent absorbance of the oligomer hybrids **1*2**–**1*6** and controls **7*2** and **7*6**.

7. UV–vis melting curves of hybrid 1*7 with non-complementary DNA sequences

The temperature-dependent absorbance of the two oligomers with a non-complementary DNA sequence does not produce a sigmoidal melting curve and the hyperchromicity is rather low, indicating that no duplex is formed.

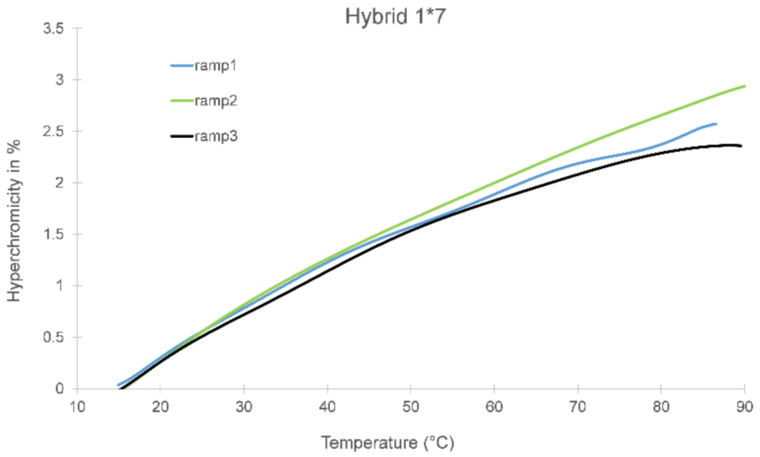

Figure S12: Temperature-dependent absorbance of the oligomer hybrid **1*7**. The T_m was recorded in a cooling (ramp 1) – heating (ramp 2) – cooling (ramp 3) mode using 0.3 °C/min, monitoring at 260 nm.

8. References

[1] H. Bittermann, D. Siegemund, V. L. Malinovskii, R. Häner, *J.Am.Chem.Soc.* **2008**, *130*, 15285–15287.

[2] N. Rahe, C. Rinn, T. Carell, *Chem.Commun.* **2003**, 2120–2121.
